# Pathways to improve health information systems in Ethiopia: current maturity status and implications

**DOI:** 10.1186/s12961-022-00860-z

**Published:** 2022-06-29

**Authors:** Amanuel Biru, Dawit Birhan, Gemechis Melkamu, Abebaw Gebeyehu, Afrah Mohammedsanni Omer

**Affiliations:** 1 Ethiopia Data Use Partnership (DUP), Addis Ababa, Ethiopia; 2 Ethiopia Ministry of Health, Addis Ababa, Ethiopia

**Keywords:** Health information system, Maturity assessment, SOCI tool, MEASURE Evaluation

## Abstract

**Background:**

To achieve national and international strategic goals, countries are advised to assess the maturity status of their health information systems (HIS), including business continuity and interoperability. This work aims to determine the existing maturity status of the Ethiopian HIS, set HIS improvement goals, and inform a path towards an improved national HIS by the end of 2024.

**Methods:**

This assessment was a collaborative and transparent process that was carried out with the engagement of all key stakeholders through consultation. We used the Stages of Continuous Improvement (SOCI) tool to guide the assessment to measure the maturity level of the Ethiopian HIS in five core domains, 13 components and 39 subcomponents and to guide future plans.

**Results:**

The overall average score of the national HIS maturity was 2.68/5, which is categorized between repeatable (stage 2) and defined (stage 3) maturity levels. The assessment findings revealed that three out of the five HIS maturity domains were at a repeatable stage. Only the leadership and governance and the data quality and use domains were at the defined maturity level. A majority (7/13) of the subcomponents were at the repeatable level of maturity, while four were at the defined level. Policy, legal and regulatory framework and compliance from the leadership and governance domain and interoperability from the data quality and use domain were categorized as having an emerging status. Considering the current HIS maturity status, gaps and strengths identified, ongoing HIS initiatives, existing platforms, and the interest and level of engagement of senior government leadership, this assessment put forward an improvement roadmap for achieving the desired managed stage (4.37) of maturity by the end of 2024.

**Conclusions:**

The findings show that the overall maturity level of the Ethiopian HIS is 2.68, which is between the repeatable and defined maturity stages. Enforcement of policies and legislation, data exchange among systems, and information and communication technology infrastructure business continuity planning are the main challenges of Ethiopian HIS requiring further investment. Strengthened and collaborative effort is critical to reaching the desired goal of “managed” HIS (stage 4) in the country by 2024.

**Supplementary Information:**

The online version contains supplementary material available at 10.1186/s12961-022-00860-z.

## Background

Reliable health information is the foundation of decision-making across all health system building blocks [1–3]. Striving to tap into the huge benefit and positive impact of the health information system (HIS), the Ethiopian Ministry of Health (MoH) identified digital transformation and its governance as one of the major pillars of its Health Sector Transformation Plan II (HSTP-II, 2020–2025) [4]. The MoH also developed the Information Revolution (IR) Roadmap [5] to provide overall guidance, including digital health investment, towards ensuring the availability of strong digitized HIS in the country.

In order to advance health system capabilities, countries are advised to assess the maturity status of their HIS, including business continuity and interoperability [6]. Assessing the maturity level of the digital HIS will provide information for policy-makers and implementers to understand its areas of strength and those areas that need more attention. It can also guide a strategically linked improvement roadmap [6]. If applied regularly, maturity assessment results can drive improvements in HIS that will eventually lead to better health outcomes [6].

The importance of assessing the maturity level of HIS endeavours using maturity model-based assessment tools has increased with the continuous advancements in health technology [7–10]. These methods are useful to describe the current maturity level of the HIS in terms of human resources, business processes, technology and organizational capabilities. The methods also facilitate users’ ability to set goals for future levels of maturity and inform the development of improvement plans [7–10]. These will help to realize the next maturity level towards a stronger HIS, enabling a country to meet its public health goals. HIS maturity assessments give due emphasis to the institutional maturity of the information system in its entirety, as well as the maturity of individual HIS components and the interoperability of those systems [6]. WHO, through its engagement with the country offices, assesses and publishes the HIS maturity status of countries in the form of the Global Digital Health Index [11]. Countries such as Kenya [9], Uganda [12] and Tanzania [13] likewise use maturity assessment tools to measure the maturity of different aspects of their HIS.

Over the past several years, Ethiopia has demonstrated progress in efforts towards an optimized HIS by producing guidelines and governance documents, implementing electronic HIS such as District Health Information Software 2 (DHIS2), and engaging in capacity-building and using data for planning and decision-making. However, the overall maturity of those systems and their level of interoperability has yet to be measured.

The maturity assessment reported herein is the first of its kind in Ethiopia. The assessment was aimed at determining the current maturity status of the Ethiopian HIS and its digital health progress in terms of leadership and governance, management and workforce, HIS information and communication technology (ICT) infrastructure, standards and interoperability, and data quality and use. Moreover, it aimed to identify interventions that are critical to achieving mature and interoperable HIS, and set forth an improvement roadmap describing the desired goal (by the end of 2024) of a resilient HIS in Ethiopia. The findings and recommendations of the current assessment have served as a basis for the development of the Digital Health Blueprint (DHBp), which the Ethiopian MoH launched in September 2021 [14] and will hopefully continue to serve the same for the subsequent strategies in the country.

## Methods

This assessment was a collaborative and transparent process that was carried out with the engagement of all key stakeholders through consultation and review of documents to measure the maturity level of the Ethiopian HIS and define future pathways to improve the system. Below are the steps the assessment followed.

### Establishing a team of experts

Stakeholder mapping was done with the participation of all relevant stakeholders, including directorates of the MoH, agencies under the MoH, regions in Ethiopia, implementing and funding partners, and universities, to ensure representativeness. A team of experts from the Ethiopia Data Use Partnership (DUP) was delegated to lead the HIS maturity assessment.

### Defining the scope of the assessment

This HIS maturity assessment aims to evaluate the maturity level of the overall HIS at the national level based on the HIS domains and subdomains. The assessment was conducted with the understanding that such knowledge would serve as a foundation and provide information for other HIS/digital health initiatives. Measuring individual-level HIS maturity status, however, was not within the scope of this assessment.

### Document review and selecting the assessment tool

At the initial phases of this task, experts conducted a review of the available HIS/digital health maturity assessment tools and results for different countries. They also reviewed documents, including national documents, to help them reach consensus in selecting the assessment tool, identify the strengths and gaps in the Ethiopian HIS, and suggest interventions. These documents included digital health policies, global and domestic digital strategies and guiding documents, global trends, publications, research papers and assessment reports [7–10]. After this task, the team of experts decided to adopt the Stages of Continuous Improvement (SOCI) tool using the MEASURE (Monitoring and Evaluation to Assess and Use Results) Evaluation [15] to assess the maturity of the national HIS in Ethiopia. The SOCI tool was developed to help countries holistically assess, plan and prioritize interventions and investments to strengthen their HIS. The tool measures current and desired HIS status in five core domains described below across 13 components and 39 subcomponents, facilitates HIS improvement goal-setting, and maps a path towards improvement [15]. The SOCI tool, unlike other HIS assessment tools, addresses the specific challenges that low- and middle-income countries experience in collecting and using health information and offers guidance that defines a progression to improvement in HIS [16]. This tool was implemented in Uganda to serve a similar purpose as in this research [12]. The five core domains are as follows:

1. HIS leadership and governance: deals with improving the impact of quality deliverables and organizational efficiency towards building strong governance on data quality, data management, data-sharing, data use, privacy and security, and business process continuity.
2. HIS management and workforce: addresses the availability of adequate personnel with characteristics, attributes and capabilities to perform tasks to achieve the intended goals.
3. HIS ICT infrastructure: deals with the implementation of required technology by applying standard operating procedures to enhance the daily business of the health sector and its stakeholders.
4. HIS standards and interoperability: deals with the realization of a health data exchange using nationally and internationally known and accepted standards.
5. Data quality and use: addresses data quality and data use culture-related issues for informed decision-making through well-organized systems and standard methods and techniques.

### Expert consultation workshop to define HIS maturity level and map a path towards improvement

A 7-day consultative workshop was conducted with the participation of 41 senior experts from the identified stakeholders. These experts were further categorized into five groups, each consisting of an average of eight individuals with diverse expertise.

During the first day of the workshop, participants reached a common understanding of the assessment tool and its domains, components and subcomponents; discussed the major national digital health initiatives; shared available documents; and built consensus on the scoring mechanisms.

Workshop days 2–4: Guided by the SOCI assessment tool, the team of experts worked together to define the current status of the Ethiopian HIS. The assessment followed a hybrid self- and facilitator-administered approach in which external experts from the Ethiopia DUP spearheaded the exercise. This approach is recommended by the MEASURE Evaluation to minimize self-reporting bias and documentation burden and to maximize local ownership and objectivity [15].

Although defining individual-level HIS maturity was beyond the scope of this assessment, the digital health systems, implemented and owned by the MoH, regional health bureaus and agencies were given due consideration during the process. Each domain was evaluated in light of the HIS/digital health enhancement efforts made from service delivery points (SDPs) all the way to the national level.

The maturity level was measured across five stages—emerging, defined, repeatable, managed and optimized—based on the SOCI tool (Table 1). These five stages were given a score ranging from 1 to 5, respectively. All five groups of experts completed the HIS assessment Excel worksheet through consultation among the team members and explained their decision to the whole panel. Disagreements in scoring among the teams were solved by consensus. The average score of the five groups was analysed to present a cumulative score of the current maturity level for each SOCI subcomponent. The average score of the subcomponents was taken as the total score for each component and the average score of the components was taken as the total score for each domain. The team also outlined areas of strength and major gaps in Ethiopia’s HIS under each domain, component and subcomponent.

**Table 1:** Description of the five stages of continuous improvement

Stage	Description	Score
Emerging/ad hoc	Formal processes, capabilities, experience, or understanding of HIS issues/activities are limited or emerging Formal processes are not documented, and functional capabilities are at the development stage Success depends on individual effort	1
Repeatable	Basic processes are in place based on previous activities or existing and accessible policies The need for standardized processes and automated functional capabilities is known There are efforts to document current processes	2
Defined	There are approved, documented processes and guidelines tailored to HIS projects or activities There is increased collaboration and knowledge-sharing Innovative methods and tools can be implemented and used to extend functional capabilities	3
Managed	Activities are under control using established processes Requirements/goals have been developed, and a feedback process is in place to ensure that they are met Detailed measures for processes and products are being collected	4
Optimized	Best practices are being applied, and the system is capable of learning and adapting The system uses experiences and feedback to correct problems and continuously improve processes and capabilities Future challenges are anticipated, and a plan is in place to address them through innovation and new technology Processes are in place to ensure review and incorporation of relevant innovations	5

Workshop days 5–7: The team of experts evaluated the implications of the current maturity status of the country and document reviews to identify possible interventions and set forth a roadmap outlining the desired goal of the HIS maturity level in Ethiopia. The improvement roadmap was outlined for each domain of the SOCI tool. Future desired maturity levels were also determined by taking the average score of the five groups similarly to how the current maturity level was measured.

## Results

A total of 41 experts from diverse organizations participated in this exercise to assess the maturity level of the Ethiopian HIS and set forth a roadmap to improve the system. The experts included health informatics/digital health specialists, public health specialists, HIS analysts, health policy and governance analysts, monitoring and evaluation (M&E) and research advisors, information technology specialists (digital infrastructure-affiliated), e-health architecture and systems interoperability specialists, and health system researchers. Details of the participants’ organizations are described in Table 2 below.

**Table 2:** Details of participants and respective organizations

Organization	No. of participants
MoH	9
MoH agencies	8
Regional health bureaus	6
Implementing partners and donors	14
Universities	4
Total	41

### Maturity level of the Ethiopian HIS

**Fig. 1:**
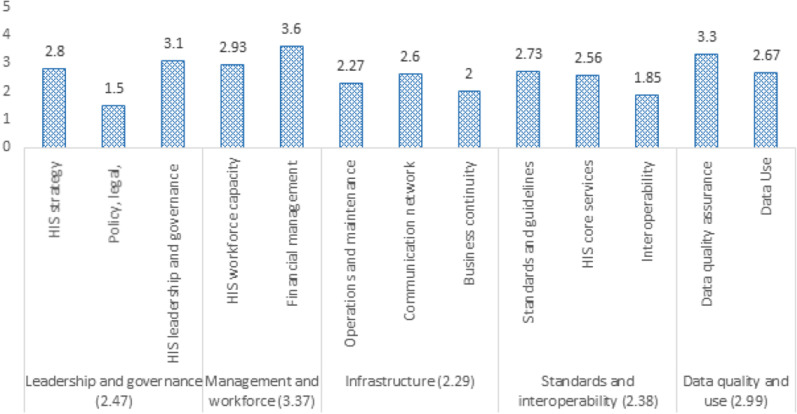
Current status of Ethiopian HIS categorized under SOCI domains, June 2021

The overall average score found for the national HIS maturity was 2.68, which is categorized between the repeatable (stage 2) and defined (stage 3) maturity levels. The assessment findings revealed that three out of the five HIS maturity domains were at a repeatable stage. Only the leadership and governance and data quality and use domains were at the defined maturity level. A majority (7/13) of the subcomponents were at the repeatable level of maturity, while four were at the defined level. Policy, legal and regulatory framework and compliance from the leadership and governance domain and interoperability from the data quality and use domain were categorized as having an emerging status (Fig. 1).

**Fig. 2:**
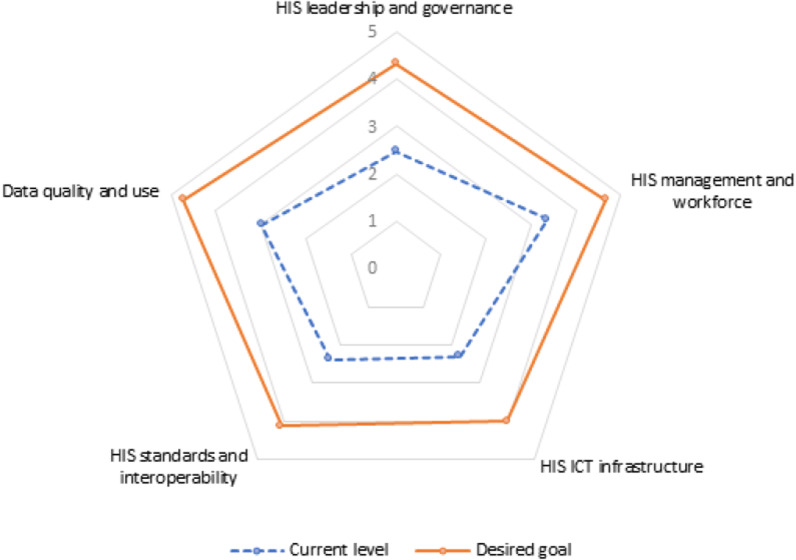
Current (2021) and desired (2024) maturity status of the Ethiopian HIS

In addition, this assessment identified major areas of strength and gaps for each SOCI domain and subdomain. Details of the identified strengths and gaps are presented in Additional file 1. Although there is a contextualized HIS strategic plan in the country, the plan is not updated or comprehensive, and has yet to be endorsed. Training and education programmes are not being reviewed on a regular basis by the designated authority to ensure sufficient HIS workforce and alignment with the evolving HIS needs and technology. Moreover, data exchange standards are not developed, no exchange standards between commodity management and HIS are established, and such exchange is not integrated into the national HIS plan. A national coordinating body (performance monitoring team, PMT) is not conducting regular data quality checks, data reviews and audits are not automated and are not analysed as required, and metrics reported on data quality issues are not used for continuous improvement. The team of experts also identified interventions that could facilitate mature and interoperable HIS, and put forward an improvement roadmap describing the “desired goal” (by the end of 2024) towards a resilient HIS in Ethiopia (Fig. 2).

The roadmap with possible interventions was developed considering the current HIS maturity status, gaps and strengths identified, ongoing HIS initiatives, existing platforms, and the interest and level of engagement of senior government leadership (Table 3). Some of the interventions include senior management endorsement and enforcement of HIS policies and legislation, developing tailored competency enhancement training and development programmes for the HIS/digital health workforce, defining the minimum national clinical data sets based on international standards, and organizing regular/consistent data quality reviews and audits and developing and using guidelines on data use impact.

**Table 3:** Summary of current maturity status (2021), identified gaps, desired goals and improvement roadmap (2024) of the Ethiopian HIS

Domains^a^	Identified gaps	Identified interventions
Leadership and governance Current maturity level = repeatable (2.47) Desired goal (2024) = 4.33	HIS strategic and M&E plans are not comprehensive, up-to-date and endorsed There is no clear structure, process or specific mechanism to address noncompliance, no law enforcement to ensure adherence to organizational policies and procedures HIS functions are not uniform across national and subnational levels, and there is no clear career path that serves for all concerned levels	Senior management buy-in, endorsement and enforcement of HIS policies and legislation Strengthening structures, processes and specific mechanisms for enforcement of policies Inclusive coordination mechanisms
Management and workforce Current maturity level = defined (3.37) Desired goal (2024) = 4.67	Informatics and project management concepts are used only in limited settings (and only in a few projects at the national level) for developing, implementing and managing HIS activities and projects Training and education programmes are not being reviewed on a regular basis by the designated authority to ensure sufficient HIS workforce and to align with the evolving HIS needs and technology Financial planning is not done in a holistic way for the entire HIS implementation life cycle, and there is a lack of consideration of HIS investments for different healthcare priorities that include sustainability. The resource mobilization plan is not periodically reviewed/revised to accommodate financial requirements needed to support evolving HIS activities and emerging health sector needs at the appropriate level of implementation (national, subnational). At the same time, there is no or very limited private–public partnership for funding of HIS implementation	Mainstreaming the informatics concept—including in academia Clear health information technology/HIS structure and incentive mechanisms Tailored competency enhancement training and development programmes for HIS/digital health workforce Assessing and deploying the HIS/digital health workforce to meet the growing demands
ICT infrastructure Current maturity level = repeatable (2.29) Desired goal (2024) = 4	Lack of an alternative power source and business continuity plan (BCP) related to power supply; lack of a responsible body that will consistently follow up on power failure issues in most of the health institutions Poor engagement of the private sector in laying out network infrastructure at public health facilities Poor coordination between internet service provider and health sector benefactors Lack of national technical standard/specifications for hardware required for HIS implementation Lack of regular network and internet connectivity assessment and reporting methods Lack of standard BCP; the existing business plans are not documented	BCP/policy—particularly to address issues of sustainable power sources, connectivity infrastructure and hardware demands Speeding up the pace of the HealthNet scale-up and maintenance Creating strong collaboration with the service providers (particularly with private sector and telecom service providers) Mobilizing resources to address the increasing hardware demands
Standards and interoperability Current score = 2.38 Desired goal (2024) = 4.11	HIS data standards and guidelines are not endorsed Data exchange standards are not developed; no exchange standards between commodity management and HIS are established, and such exchange is not integrated into the national HIS plan Limited implementation and utilization of core registry services Lack of a unique person identification system	Implementing secure data exchange, messaging and terminology standards Defining the minimum national clinical data sets based on international standards Implementing and utilizing core registry services (e.g. Master Facility Registry, Master Patient Index, Provider Index) Coordinating and working with stakeholders (e.g. Immigration Nationality and Vital Events Agency [INVEA]) on unique person identification system
Data quality and use Current score = 2.99 Desired goal = 4.72	Data reviews and audits are not conducted on a regular basis using automated and manual data quality assessment (DQA) processes to ensure defined levels of quality. Limited use of metrics reported on data quality issues for continuous improvement. DQA plan is not periodically reviewed by the coordinating body to meet the evolving data quality needs No standard operating procedures for data management integrated with the national HIS plan The data use strategy is not adapted to meet emerging decision-making needs of programme managers, policy-makers and providers interacting with HIS Condition-specific order sets and documentation templates are not defined; knowledge-based systems are not implemented in some settings to support decision-making The data systems/applications in use do not fully ensure reliable and appropriate access to data at all levels for authorized users; changes in reporting requirements cause significant disruptions to data availability Data use competency development is not tracked by user type and is not level-based Guidance on the design and use of information products is not up to date, implemented or monitored for compliance by an established governing body Parameters for the measurement of the impact of data use are not well defined, implemented or monitored. Metrics on reporting and analysis capabilities are not used for continuous improvement	Regular/consistent data quality reviews and audits—and automating the process Dynamic data use strategy to meet the emerging decision support needs at all levels—including data use competency mechanisms Developing and managing data repositories and warehouse Standardizing the design, use and dissemination of information products Developing and using guidelines on data use impact

^a^*DQA* data quality assessment

## Discussion

This HIS assessment was conducted to measure the current maturity status of the HIS/digital health system at the national level, setting goals for the components of the system and suggesting possible interventions for continuous improvement.

The results of the current overall HIS maturity assessment indicate the status of the Ethiopian health sector in terms of the five key domain areas. Accordingly, the national HIS maturity level is between repeatable (stage 2) and defined (stage 3), with an average score of 2.68. This indicates that basic HIS/digital health processes are in place; the processes are based on existing and accessible policies; the need for standardized processes and automated functional capabilities is known and they are partly practised; there are approved, documented processes and guidelines tailored to HIS projects and activities; and a sense of collaboration and knowledge-sharing among stakeholders is increasing. This finding is in line with the results of the recent HIS maturity assessment conducted by WHO, in which Ethiopia scored a “desired” HIS maturity level [11]. Our finding is consistent with other African countries such as Cape Verde, Ghana, Mali, Nigeria and Benin, where the HIS is at a “desired” level of maturity [11].

The assessment results showed that HIS management and workforce and data quality and use domains are at the defined stage of maturity. HIS management and workforce is a key component for the health sector at large to rely on health information for evidence-based decision-making, health service planning and delivery of quality care [15]. In the Ethiopian health sector, there are well defined and documented competencies, roles and responsibilities for HIS task forces at almost all levels, even though much work has to be done in conducting regular HIS capability assessments and analyses. The country also has a multiyear HIS financing strategy [17] aligned with healthcare and HIS strategic priorities, and financial sources are identified for sustained HIS activities, which requires setting priorities in resource allocation.

Currently, Ethiopia implements data quality and use procedures with clear and defined rules for data collection, processing, analysis and use at all levels. A regular schedule is also defined for conducting data quality reviews and audits. Despite these efforts, this assessment indicates the need for a functional governing body for national data quality and use with standardized processes and engagement of health data actors. The findings also show the need to develop data quality plans that will be reviewed periodically, using defined standards and procedures by a coordinating body at all levels. Moreover, it calls for a concerted effort in nurturing a data use culture through advocacy and promotion, establishing knowledge management centres to transfer knowledge and skill, using recognition and incentive mechanisms, tracking data use impact and monitoring data use culture improvements.

This assessment also indicated that the domain leadership and governance is at a “repeatable” maturity level in Ethiopia. While promising progress has been made with regard to drafting workable documents, engaging stakeholders, and establishing technical and administrative committees, the sector in Ethiopia will have to strive to improve particular areas such as endorsing draft governance documents, defining the career path, revising the HIS structure and budgeting the M&E activities. Optimal maturity of this domain in Ethiopia will deliver operational certainty and stability focused on the goals of both the HSTP-II and DHBp, which are crucial in terms of improving the enforcement of policies, legislation and strategies for digital health; the alignment and implementation of the M&E plan; the definition of the organizational structure, coordination and functions of HIS; and setting the mechanism for HIS compliance.

Similarly, this assessment showed the need for strengthened effort to improve the maturity level of ICT infrastructure and HIS standards and interoperability domains in Ethiopia. To meet the goals of the HIS ICT infrastructure domain, MoH and its stakeholders will have to work towards ensuring stable power sources, fulfilling hardware requirements, enhancing connectivity, developing expert-level capacity and establishing business continuity at all levels. To address the current gaps, the development of harmonized and comprehensive plans is crucial.

The main gap identified regarding data exchange efforts is the lack of established HIS standards and guidelines and the need to develop a client registry service and enhance existing national registry services based on a formal feedback process. Efforts are needed to address the gaps in standards, guidelines and minimum data set development, update and maintenance, particularly in terms of establishing core repositories (such as client and provider indices) and endorsing, enforcing and monitoring the available standards from MoH and its stakeholders.

Zooming in to the individual components of each domain, we can further understand the existing challenges and where to invest in the years to come in addition to maintaining what has worked well. These areas include enforcement of policies and legislation, data exchange among systems, and ICT infrastructure business continuity plans that have “emerging” maturity levels. Addressing these will take a tailored and coordinated effort.

The HIS state targeted for 2024 (end of HSTP-II) is the “managed” (stage 4) maturity level, with an average score of 4.37. This level of maturity indicates that activities are under control, using established processes; requirements/goals have been developed and a feedback process is in place to ensure that they are met; and detailed measures for processes and products are in place and are exercised to the acceptable level.

The findings of this assessment, including the identified gaps with suggested interventions, were used to shape the national DHBp, which is now the main document for digital health investment in the country. The current HIS maturity status of the country will serve as a baseline for future assessment, and the DHBp, after its full implementation, will also be measured in terms of these domains.

## Conclusion and recommendations

Our assessment used the SOCI tool to measure the maturity level of Ethiopian HIS in five core domains, 13 components and 39 subcomponents. This study revealed that the overall maturity status of the Ethiopian HIS is 2.68, which is between a “repeatable” and “defined” maturity stage. Enforcement of policies and legislation, data exchange among systems, and ICT infrastructure business continuity plans are the main challenges of Ethiopian HIS requiring further investment. Strengthened and collaborative efforts are critical to reaching the desired goal of “managed” HIS in the country by 2024.

The digital HIS implemented thus far by the Ethiopian MoH has played pivotal roles in the improvements seen towards addressing the access, quality and usability of health and health-related data. Therefore, based on this study, individual HIS components which have a wide impact and coverage in the country should be further assessed and action plans should be set in order to address the identified gaps and execute the proposed interventions.

Moreover, as measuring the maturity level of a country’s HIS is not a one-time task, this assessment should be conducted continuously in order to have a clear understanding as to where the system stands and how it is moving towards its targets.

## Supplementary Information

**Additional file 1.** Major areas of strength and gaps of Ethiopian Health Information System, 2021.

## Data Availability

The data sets used and/or analysed during the current study are available from the corresponding author on reasonable request.

## Abbreviations

DHBp: Digital Health Blueprint
DUP: Ethiopia Data Use Partnership
HIS: Health information system
HSTP: Health system transformation plan
ICT: Information and communication technology
MoH: Ministry of Health
SDP: Service delivery point
SOCI: Stages of Continuous Improvement

## References

[1] UNDP Capacity development for Health; URL: https://www.undp-capacitydevelopment-health.org/en/capacities/focus/health-information-systems/ (Accessed: December 11, 2021)

[2] Health Information Systems, World Health Organization. 2008

[3] World Health Organization. Health Metrics Network Framework and Standards for Country Health Information Systems. 2008.

[4] Ethiopia Ministry of Health. Health Sector Transformation Plan (HSTP-II, 2020–2025).

[5] Ethiopia Ministry of Health. Information Revolution, 2016.

[6] MEASURE Evaluation and Health Data Collaborative. Health Information Systems Interoperability Maturity Toolkit: Users’ Guide.

[7] MEASURE Evaluation. Building a network: New interoperability toolkit helps integrate health information systems in developing countries. 2019. https://www.measureevaluation.org/news/building-a-network.html.

[8] MEASURE Evaluation. Building a Strong and Interoperable Health Information System for Ghana. 2018.

[9] Nyangena J, Rajgopal R, Ombech EA, Oloo E, Luchetu H, Wambugu S, Kamau O, Nzioka C, Gwer S, Ndirangu MN (2021). Maturity assessment of Kenya’s health information system interoperability readiness. BMJ Health Care Informat..

[10] Carvalho JV, Rocha Á, Abreu A (2019). Maturity assessment methodology for HISMM-hospital information system maturity model. J Med Syst.

[11] World Health Organization. Global Digital Health Index. https://www.digitalhealthindex.org/.

[12] MEASURE Evaluation. Mapping a Path to Improve Uganda’s Health Information System Using the Stages of Continuous Improvement Toolkit. Workshop Report. Chapel Hill, NC, USA: MEASURE Evaluation, University of North Carolina. (2019).

[13] https://www.measureevaluation.org/his-strengthening-resource-center/country-profiles/tanzania.

[14] Ethiopia Ministry of Health. Digital Health Blueprint (DHBp) 2021.

[15] MEASURE Evaluation. HIS Stages of Continuous Improvement (SOCI) Toolkit.

[16] Kumar M, Millar L. Stages of Health Information System. 2017.

[17] Alebachew A, Yusuf Y, Mann C, Berman P. Ethiopia’s Progress in health financing and the contribution of the 1998 health care and financing strategy in Ethiopia. MA, Addis Ababa: Harvard TH Chan School of Public Health and Breakthrough International Consultancy, PLC. 2015.

