# Supplementary material for: Pathways to improve health information systems in Ethiopia: current maturity status and implications
Source: Health Res Policy Syst. 2022 Jun 29;20:78. doi: 10.1186/s12961-022-00860-z (PMC9245200; doi:10.1186/s12961-022-00860-z)
Supplement: Supplementary file 1 — Additional file 1. Major areas of strength and gaps of Ethiopian Health Information System, 2021. [file 12961_2022_860_MOESM1_ESM.docx]

**Additional file 1:** Major areas of strength and gaps of Ethiopian Health Information System, 2021.

| **Domains** | **Findings on major areas of strength and gap** |
| --- | --- |
| **HIS Leadership and Governance**  **Maturity level** =  Repeatable (2.47) | **HIS strategy *(2.8)***  *HIS/Digital Health Strategy:* The HIS strategic plan was prepared in the context of the health priorities of the country; the strategy has a vision for management and use of health information; it contains a plan of action for delivering the vision, and arrangements for M&E; digital strategy also has been prepared to facilitate the leapfrogging to the digital health era. However, the HIS and Digital Health strategies are not yet endorsed and put into action.  *M&E Plan:* A draft framework prepared for regular evaluation (both formative and summative) of HIS activities; the M&E plan is aligned with HSTP-II and is regularly reviewed; dissemination platforms available. Nevertheless, these HIS activities are not well-aligned with the HIS strategy; M&E is not fully implemented, sufficiently budgeted, nor consistent; the M&E lacks checks and balances across all HIS systems. |
|  | ***Policy, legal, and regulatory framework, and compliance (1.5)***  *Policy, legal, and regulatory framework and compliance Existence of HIS policies and legislation:* Draft HIS policies and legislations available to guide decisions and achievements of HIS outcomes. Compliance documents are not updated and endorsed; they lack oversight for adherence and wider stakeholder engagement; duplication of policies and legislations do exist (e.g., Data Sharing Policies drafted by EPHI, MoH, and partners).  *Policy compliance enforcement:* There are specific enforcing mechanisms in some settings. For example, data sharing policies in EPHI and MoH. No clear process for the review, validation, and enforcement of policies, legislation, and regulations; no specific mechanisms and regulatory body to ensure adherence to organizational policies, procedures, and best practices related to HIS/digital health; weak accountability mechanisms; no strong metrics on compliance and noncompliance of the policies. |
|  | ***HIS leadership and governance organizational structures and functions (3.1)***  *HIS leadership and coordination:* Have established technical and administrative committee such as NAG, TWG, PMT, IR steering committee; had a meeting on different committees such as JSC (MoH with the RHBs heads) to address political issues and to manage national HIS affairs at all levels of a country’s health system; the governance structure consists of the mechanisms, processes, and institutions through which actors and stakeholders articulate their interests, by defining the roles and responsibilities with meeting schedule. The HIS leadership and coordination lack regularity to oversee the function and implementation of the HIS; high turnover at the executive leadership level; weak coordination and oversight at national-level; weak established process for sharing and reviewing HIS information with all HIS stakeholders.  *HIS organizational structure and functions:* Defined organizational structures and processes at some levels, there are job titles and clear descriptions of duties and responsibilities. However, the structures are not uniform across all levels and are not attached to an accountability framework. |
| **HIS management and workforce**  **Maturity level** = Defined (3.37) | **HIS workforce capacity and development (2.93)**  *HIS competencies (knowledge, skills, and abilities):* There are well defined and documented competencies, roles, and responsibilities for HIS task forces at almost all levels; the competencies for the HIS workforce are aligned and practiced with the HIS strategies; HIS training program courses are aligned with established core competencies to meet training needs; an established career path is defined for HITs. The informatics and project management concepts are used in limited settings (just in some projects at the national level) for developing, implementing, and managing digital health activities and project; limited HIS capability assessments and analyses; lack of a strong hiring mechanism of distributing HIS workforce to all health offices and facilities; the workforce distribution significantly varies from region to region regardless of the demand.  *HIS training and education (include continuous professional development):* Training, academic curricula, and processes for developing training and education programs to build HIS skills and competencies nationally are standardized; training and education programs conducted periodically at government- designated institutions. Clear and measurable learning outcomes are defined for training courses; training and education plans are integrated in HIS implementation plans and the results are measurable. Training and education programs are not being reviewed on a regular basis by the designated authority to ensure alignment with HIS needs and technology.  *HR Policy:* There is a structured hiring mechanism that distributes staff to some subnational facilities; HIS competencies, roles, and responsibilities of staff are clearly documented; human capacity needs are integrated in the HIS and/or health plan and monitored by a designated government authority; the HIS Workforce Analysis/Labor Market Analysis (HLMA) is conducted nationally to forecast future demands. The workforce is not sufficient to meet HIS workforce demands at health offices and health facilities; data on vacancies and staffing needs are not collected and managed in the HRIS on a regular basis nor are they used to inform hiring, distribution of staff, training and education needs, or advocating for budgets to meet national HIS needs; HIS/digital health competencies, roles, and responsibilities of staff performing HIS functions are not disseminated to the concerned staff; region specific HIS Workforce Analysis/Labor Market Analysis (HLMA) is not conducted to forecast future demands. |
|  | **Financial management (3.6)**  *HIS financing plan:* There is a multi-year HIS financing strategy aligned with healthcare and HIS strategic priorities, and sources are identified for sustained HIS activities; HIS implementation is funded using capital financing, revenue, and grants; expenditure reports are shared with the relevant HIS team/unit; financial audit processes are in place and regularly carried out to promote accountability in HIS spending; established financial management system is owned, reviewed, tracked, and revised by the government using the Integrated Financial Management System (IFMIS). Limited private-public partnership (PPP) funding for HIS implementation; lack of strategic HIS investment guidance to dictate future demands of HIS/digital health; lack of inclusiveness of the financial plans.  *Resource mobilization:* The resource mobilization plan for HIS activities is integrated in the HIS and/or health plan at the appropriate level of implementation (national, regional). The resource mobilization plan is not periodically reviewed/revised to accommodate financial requirements needed to support evolving HIS activities and emerging health sector needs at the appropriate level of implementation (national, subnational). |
| **HIS ICT Infrastructure**  **Maturity level** = Repeatable (2.29) | **Operations and Maintenance (2.27):** There is a responsible team within the Ministry that handles power management issues; there is a backup power source installed at the national level (MoH’s Datacenter), some RHBs and agencies (though not capable of handling the current demands). ICT policies/directives are prepared at Ministry and agency levels; IT Internship programs have been promoted by engaging IT professionals who are recent graduates to facilitate support and maintenance at woredas and health facilities. Lack of a sustainable/ alternative power source at most healthcare facilities; lack of a business continuity plan related to power supply in most of the health institutions; lack of collaboration between health facilities and small-scale enterprises to address maintenance and technical assistance demands at the facility level; poor planning for replacing outdated/ damaged hardware; lack of a clear plan to address the increasing hardware equipment demand at woreda and facility levels. |
|  | **Communication Network (LAN and WAN) (2.6):** There is a dedicated ICT infrastructure and virtual private network at the MoH, agencies, as well as at the regional level; HealthNet and LAN are deployed in many health facilities; enhancing the network and Internet connectivity is considered as one of the key initiatives of the Digital Health Strategy; mechanisms exist to identify challenges of connectivity (e.g., Service Availability and Readiness Assessment [SARA]). Communication gaps between the Internet service provider (Ethio-Telecom) and health institutions; inadequate follow-up and technical support from the MoH and regions in sustaining HealthNet utilization; lack of regular network and Internet connectivity assessment and reporting methods; lack of redundant Internet/WAN connection options. |
|  | **Business continuity:** To avoid business discontinuity, a backup datacenter has been implemented at the national level for key HIS. While there are some decent business continuity discussions and plans, they are not well documented, prioritized, nor endorsed; no clear BCP for health facilities; no business continuity standard procedures thus far. |
| **HIS Standards and Interoperability**  **Maturity level** = Repeatable (2.38) | **Standards and Guidelines (2.73):** All data sets are developed in-line with national guidelines; the indicator set is integrated into the national health strategy; aggregated data sets are harmonized/mapped with those from internationally recognized standards; there is a recognized need for a harmonized data exchange of HIS and better coordinated stakeholder initiatives; national Indicator Reference Guidelines, national health data dictionary (NHDD), MFR guidelines, and data management guidelines are centrally prepared. Foundational standards and guidelines have been developed but not endorsed by MoH, which hinders the adoption and practicality of the standards; clinical minimum data sets are not developed; standards for data exchange/messaging are not yet prepared. |
|  | **HIS Core Services (2.56):** The MFR system is at a final stage to be operational and much effort is needed to scale it up at a national level; the national digital health strategy has identified leading indicators to monitor progress and is being implemented using national HMIS/DHIS2; NHDD terminology has been introduced using a mobile application for collection, dissemination, and use of the terminologies; efforts to access metadata are consolidated and available from a single portal; national health information architecture is up-to-date and being implemented and includes foundational interoperability tools required to perform HIS functions. Registry services are foundational for other health data exchange and harmonization, but there is a limitation in the regular update and feedback process of the implemented core services; a client registry is not developed and lack of a national digital ID has made it hard to do so. |
|  | **Interoperability (Data Exchange) (1.85):** Though data exchange implementation is not at a large scale, it has been localized and ad hoc efforts are observed. Interoperability between DHIS2 and MFR is a pilot stage. Some essential shared services, such as unique person identification, are missing and that hinders a national-level person data exchange; aggregate data exchange practices exist but are on a limited scale; there are no security standards for the data exchange implemented; national interoperability LAB does not exist to test and there is no certification for implementers to stick to. |
| **Data quality and use**  **Maturity level** = Defined (2.99) | **Data quality (DQ) assurance (3.3)**  *DQ assurance and control*: Procedures for data collection, processing, analysis, and use are defined and implemented at all levels; a regular schedule is defined for conducting data quality reviews and audits; a national coordinating body (PMT) was established to oversee data quality; there are procedures for documenting metadata (indicators, data elements, data set, registers, tally sheets). A national coordinating body (PMT) is not doing regular data quality checks; data reviews and audits are not automated and are not analyzed as required; metrics reported on data quality issues are not used for continuous improvement; the data quality assurance plan is not periodically reviewed by the coordinating body to meet the evolving data quality needs; standards are not sufficiently used to implement data exchange and to avoid double-entry.  *Data management:* Data management processes (timely data collection and reporting, analytics and visualization) are implemented and monitored for compliance; HMIS standards (data quality and data use guides, recording and reporting guides, and indicators reference guides) are available both in electronic and manual formats. The standard operating procedures for data management are not integrated with the national HIS plan; data quality is not actively monitored and shared with stakeholders. |
|  | **Data use (2.67)**  *Data use strategy:* An integrated data quality and data use PMT platform and governance body has been established and documented. Implementation of the data use strategy is not monitored, reviewed, and given proper oversight by the established governing body; relevant data are not consistently shared with stakeholders; the data use strategy is not adapted to meet emerging decision-making needs of program managers, policymakers, and providers interacting with HIS, such as quality improvement projects, equity indicators, etc.  *Information/Data availability:* Data systems/ sources (routine, population-based) are clearly defined, designed, and implemented to support longitudinal availability of health data. Not all required stakeholders are accessing the data they require; data availability is not monitored for continuous improvements and to meet emerging health sector needs.  *Data use competencies:* Data use competencies are defined, up-to-date, and integrated in training courses (both in in-service and pre-service). Data use competency development is not tracked by user type and is also not level-based; there is no standardized plan for tracking and measuring competencies; though there are a few start-up efforts, there is no established mechanism to reward data use.  *User/ Stakeholders Engagement:* The HIS stakeholders engagement strategy is available; guidance — PMT for users’ engagement is documented and available.The guidelines for stakeholders’ engagement is not periodically reviewed and revised to address emerging and future decision-making needs of users.  *Data synthesis and communication:* Guidance on the design and use of information products (monthly analytical reports, Annual and Semi-annual Review Reports, Joint Steering Committee Report, Annual Special Bulletin, KP Dashboards, etc.) - is documented and availed. Guidance on the design and use of information products is not monitored for compliance by an established governing body, and it is not periodically reviewed and revised to ensure its applicability and relevance to emerging and future decision-making needs; no clear guidance is available for the design and use of advanced analytics.  *Reporting and Analytics feature:* Established national systems and guidelines to support standardized routine reporting; automated data reporting from point of service to national systems (via the likes of DHIS2, eCHIS); basic reporting and analysis features within applications (e.g., DHIS2, eCHIS). Lack of consistency in the use of metrics on reporting and analysis capabilities; equity issues (across regions of the country) in terms of automating and implementing data reporting tools from point of service to national systems.  *Data use impact:* Parameters on the measurement of the impact of data use are defined nationally. Parameters on the measurement of the impact of data use are not integrated in the HIS and/or health plans, and are not monitored and documented.  *Data collection alignment:* Some healthcare workflows are documented and are aligned with data collection processes; some capability to reuse collected data within a documented workflow exists locally. Technology applications from different entities may not serve a common goal and are not linked and exchanging data; HIS applications often do not comply with the country’s interoperability plan; only limited capabilities exist to reuse collected data and resources seamlessly within the workflows (particularly the gap is huge at service delivery levels).  *Decision support:* Decision support tools exist in some settings and are based on alerts and reminders to the program managers, care providers, and patients; there is a recognized need and motivation to establish standard procedures to support decision- making. Lack of decision support tools that incorporate program and clinical guidelines; lack of well-defined condition-specific order sets and documentation templates to facilitate decisions; lack of knowledge management systems. |
